# Supplementary material for: National snakebite project on capacity building of health system on prevention and management of snakebite envenoming including its complications in selected districts of Maharashtra and Odisha in India: A study protocol
Source: PLoS One. 2023 Feb 17;18(2):e0281809. doi: 10.1371/journal.pone.0281809 (PMC9937454; doi:10.1371/journal.pone.0281809)
Supplement: S3 File — (PDF) [file pone.0281809.s003.pdf]

ICMR- NATIONAL INSTITUTE FOR RESEARCH IN REPRODUCTIVE AND CHILD HEALTH

J. M. Street, Parel, Mumbai 400 012

Informed Consent Form

I ________________________________ have read / have had read the participant information sheet version no. 1.2 dated 01.09.2021 bearing page numbers 1-8 of the research study entitled ***"ICMR National Snakebite Project (INSP) on capacity building of health system on prevention and management of snakebite envenomation including its complications"***.

The information contained in the participant information sheet regarding the nature and purpose of the study, safety, and its potential risks / benefits and expected duration of the study, and other relevant details of the study including my role as a study participant have been explained to me in the language that I understand. I have had the opportunity to ask queries, which have been clarified to my satisfaction.

I understand that my participation is voluntary and that I have the right to withdraw from the study at any time without giving any reasons for the same. This will not affect my further medical care or any legal rights.

I understand that the information collected about me during the research study will be kept confidential. The representatives of sponsor, government regulatory authority's / ethics committees may wish to examine my medical records / study related information at the study site to verify the information collected. By signing this document, I give permission to these individuals for having access to my records.

I hereby give my consent willingly to participate in this research study. I am informed that I will not be given any compensation/ reimbursement for participation in the study.

For limited readers or non-readers (Illiterate participants): I have witnessed the consent procedure of the study participant and the individual has had the opportunity to ask questions. I confirm that the individual has given consent freely.

Signature of an impartial witness<br>with date<br>(Only if the participant is illiterate)	Signature/Thumb impression of<br>study participant with date
Name of the Witness	Name of the study participant
Signature of PI with date	Signature of person administering<br>the consent with date
Name of the PI	Name of the person administering<br>the consent
