# Supplementary material for: National snakebite project on capacity building of health system on prevention and management of snakebite envenoming including its complications in selected districts of Maharashtra and Odisha in India: A study protocol
Source: PLoS One. 2023 Feb 17;18(2):e0281809. doi: 10.1371/journal.pone.0281809 (PMC9937454; doi:10.1371/journal.pone.0281809)
Supplement: S5 File — (PDF) [file pone.0281809.s005.pdf]

RESEARCH PROTOCOL

Title of the Study: ICMR National Snakebite Project (INSP) on capacity building of health system on prevention and management of snakebite envenomation including its complications

Study duration: 2 years

Objectives of the study:

I. To increase the awareness and empower the community on prevention, first aid and early transport of snakebite patients to nearest health facility for reducing the mortality and morbidity due to snakebite envenomation in selected districts of Maharashtra and Orissa
II. To evaluate the component of training of the Medical Officers and other healthcare providers on management of snakebite and also understand the ASVs distribution and utilization (availability, factors restricting availability, quantity) in public health facilities in selected districts of Maharashtra and Orissa
III. To empower the health system for management of snakebite envenomation and optimal utilization of Anti snake venoms (ASVs) through implementation of Standard Treatment Guidelines (STG) of Government of India
IV. To study the impact of capacity building of health system on reducing the mortality and morbidity due to snakebite envenomation in selected districts of Maharashtra and Orissa

Introduction

Snakebite envenoming is a neglected tropical disease (NTD) resulting from injection of venom (mixture of highly specialized toxins) by venomous snake into humans usually under accidental circumstances. Snakebite envenoming most commonly affects poor, agricultural and migrant workers, tribes, hunters, often earning member of their families, and thus it is not only a public health problem, but also a major socioeconomic challenge especially in India. Globally, around 81,000 to 138,000 deaths were estimated resulting from 1.8 million to 2.7 million cases of snakebite envenoming and leading to around 400 000 people with permanent physical or psychological disabilities including blindness, amputation, and post-traumatic stress disorder [1]. However, the snakebite burden is likely to be an underestimate as snakebite is not a notifiable disease and many bites and deaths go unrecorded. The death and disability burden of snakebite is equivalent to that of prostate or cervical cancer, and is greater than any other neglected tropical disease. [2] Despite this, it has been a low priority to the, research funding agencies, health authorities and pharmaceutical companies worldwide.

According to National mortality survey (2001-2003), India constitutes almost half of the global snakebite burden with estimated mortality ranging from 45,000 to 50,000 every year [3]. This study

also reported higher annual age-standardized mortality rates per 100,000 population due to snakebite in 13 states : Andhra Prades (6.2), Madhya Pradesh (5.9), Odisha (5.6), Jhrakhand (4.9), Bihar (4.9), Tamilnadu (4.7), Uttar Pradesh (4.6),Chhatisgarh (4.4), Karnataka (4.2), West Bengal (3.5), Gujarat (3.5), Rajasthan (3.3), Maharashtra (3.0) [3]. Jharkhand and Orissa states had higher number of deaths at ages 5–14 years whereas deaths at older ages were prominent in Andhra Pradesh, Bihar, Madhya Pradesh, and Uttar Pradesh. Female deaths were more than male deaths due to snakebites in Bihar, Madhya Pradesh, Maharashtra and Uttar Pradesh [3]. There is huge gap between number of snakebite deaths reported from direct survey and hospital based data. Only 7.23 % snake bite deaths were officially reported [4].

Most of the people are bitten in rural and/ tribal areas during working in agricultural fields, fetching drinking water, sleeping on floor, going to school, or walking to an outdoor toilet without foot wares [2]. Community education is therefore crucial, because many snakebites are not reported or people seek care too late. Most of these are deaths are preventable. The community is not aware about the occupational risks and simple, cost effective preventive measures can prevent a bite. There is a need to increase community awareness on prevention of snakebites, bring behavioral changes regarding occupational risk and its reduction and empower the community on first aid skills and early transfer of snakebite patients to nearest health facility to avoid delay in ASV administration to improve the outcome. The community is also required to be educated on several do’s and don’t s regarding first aid for snakebites as recommended by National snakebite management protocol (2009) as well as STG, 2017.

There are about 236 species of snakes in India, most of which are non-venomous. However, 13 known species are venomous. The venomous snakes found in India belong to three families Elapidae, Viperidae and hydrophidae (Sea Snakes). The most common Indian elapids are Naja naja (Indian Cobra) and Bungarus caeruleus (Indian Krait), Daboia russalie (Russells’ Viper) and Echis carinatus (Saw scaled viper).[5,6] There are regional differences in the snakebite species reported in different parts of India. [7] Currently, treatment quality is highly variable from good quality in some areas to very poor in other areas. The high fatality due to Krait bite is attributed to the non-availability of anti-snake venom (ASV), delayed and inappropriate administration of ASV, lack of standard protocol for management and inexperienced doctors and ventilator or bag and valve [8]. In India, there has always been a crisis of antivenom supply [9].

The Snakebite envenoming was classified by WHO as a high-priority neglected tropical disease In 2017, and subsequently in May, 2018, Seventy-first World Health Assembly (WHA) adopted a resolution that provides a strong mandate to WHO for global actions on reducing the burden of snake bite envenoming[10]. Recently, on May 23, 2019 at the World Health Assembly, WHO launched its roadmap with an aims to halve death and disability from snakebite by 2030 [2]. The overall strategy focuses on prevention of snakebite; provision of safe and effective treatment; strengthening health systems; and increased partnerships, coordination, and resources. Community education and accelerating development of antivenom, stockpiling anti-venoms, and stabilizing the market for snakebite treatments are also recognized as important aspects in the strategy [2,11].

The Director General of Health Services, Ministry of Health & Family Welfare, Government of India released National Snakebite Management Protocol in 2009. Subsequently, Ministry of Health & family Welfare (MoHFW), Government of India through National Health System Resource Centre (NHSRC) has developed Standard Treatment Guidelines, (STG, 2017). The guidelines covers all clinical issues and aspects of management of snakebite including clinical features, community interventions measures (first aid, transport and referral criteria), snakebite diagnostic features, treatment with anti-snake venom, snakebite complications and snakebite management in primary, community/dispensary health care centres up to tertiary care including follow-up and rehabilitation of the snakebite victims. The guidelines also cover all populations and age groups including pregnant women. These guidelines are now available on the website of MoHFW.

In India, especially in primary health centers, the medical doctors are replaced every 6–12 months either due various reasons and reported to have poor knowledge about, and experience in, management of snake bites [6]. Similarly, multiple protocols are followed at secondary and tertiary level health facilities mainly from western textbooks that are not appropriate for our Indian settings. There is no formal training of medical students and/or interns on snakebite management in India. There is no formal training program for Medical Officers working in public health system in India. The insert of Indian polyvalent anti-snake venoms manufactured in India also need to be updated as per the Standard Treatment Guidelines, Government of India on recommended ASV dosage and also removal of ASV skin testing from the insert is urgently required.

WHO's strategy of reducing the number of snakebite-related deaths and disabilities by 50% by 2030 is achievable, as they are mostly preventable. However, this requires a strong commitment

and coordination at different levels specifically Ministry of Health and Family Welfare, Government of India, Department of Health Research, Indian Council of Medical Research ministry and State Health Authorities in Indian states having high burden of snakebites.

ICMR- National Task Force Expert Group Meeting for " Research on snakebite in India" held on 6th August 2019 recommended to develop project on Health System Research to study the issues and components of the training of the doctors and paramedics for the proper management of snakebite and optimal utilization of ASV, IEC for increasing awareness at the community level for the better management and prevention of snakebites.

Based on the recommendations of the ICMR- National Task Force Expert Group Meeting the aim of the proposed study is capacity building of health system on prevention, and management of snakebite as per the Standard Treatment Guidelines, optimal utilization of ASV and empowerment of the community for improved management and reduce the mortality and morbidity due to snakebites in India.

Preliminary work done :

- Much before the World Health Assembly (WHA) resolution (2017) and WHO strategy (2019) to combat snakebite, Department of Health Research and Tribal Health Research Forum of ICMR funded a pilot study on community empowerment and capacity building of public health system on snakebite management in Dahanu block, Maharashtra State through Model Rural Health Research Unit (MRHRU). In our previous study, we investigated incidence, management practices of snakebites at Sub District Hospital, Dahanu, Maharashtra based on retrospective data and reported 4.5 % case fatality rate due to snakebite envenomation at First Referal Unit (FRU) in Dahanu. There was lack of awareness amongst the Medical Officers and other health care providers about National Snakebite Management Protocol (2009) and Standard Treatment Guidelines (STG,2017) for management of snakebite envenomation (Gajbhiye et al., 2019). There was no uniform protocol followed by the Medical Officers for management of snakebite envenomation cases at first referral unit (FRU), Sub District Hospital Dahanu.[13]

- ASV skin testing was observed as a common clinical practice in tribal block of Dahanu, Maharashtra, India leading to wastage of precious ASVs. [14] (Moreover, we observed that majority of the medical officers posted at PHC do not treat the snakebite patients mainly due to fear of anaphylaxis and lack of confidence in ASV administration.

- Training manuals were prepared in local language (Marathi) for ASHA, ANM, MPW on prevention, diagnosis and management of snakebite based on the standard treatment guidelines (STG, 2017) developed by Government of India.
- IEC materials were developed in local language (Marathi) for empowerment of the community and primary health care system to empower them on snakebite prevention, diagnosis and management.
- The IEC materials were displayed at Schools, Gram Panchayats, anganawadi, PHCs, RH, SDH, Ashram Schools, Panchayat Samiti.
- Capacity building of outreach health workers (ASHA,ANM and MPW) was carried out

- The capacity building of the Medical Officers in entire Dahanu block was carried out through regular training programs and standard treatment guidelines (STG, 2017) for snakebite management was implemented. Dr Himmatrao Bawaskar, an expert on snakebite and scorpion sting envenomation conducted the training programs.

The community empowerment through culturally appropriate IECs, capacity building of health system and implementation of Standard Treatment Guidelines of Government of India for snakebite treatment in a limited setting of Dahanu block in Palghar District of Maharashtra State has shown improved outcomes as evident from reduction of case fatality rate from 4.4 % to 0.4 %.

Based on the study conducted in Dahanu block of Palgha District, Maharashtra, a multi-sectoral approach of community awareness, mapping of vulnerable populations, capacity building of health care facility, empowerment of health care workers (HCWs) was proposed for reducing the mortality and morbidity due to snakebite envenoming in India. [15]

Highlights of the impact of the study:

Parameters	Before Capacity Building	After Capacity Building
**Incidence**	Incidence of Snakebite + unknown bite 216 (2013), 264 (2014), 338 (2015) per 1Lakh population	Incidence of Snakebite + unknown bite 304 per 1 Lakh population (June 2017- May 2018)
**Case Fatality Rate**	Overall Case Fatality Rate (2014-16) : 4.4 %	Reduction in Case Fatality Rate - 0.4%

**Awareness and Training**	No prior training on snakebite and scorpion sting management to MOs<br><br>No awareness on Snakebite management guidelines (2009) and STG (2017) of Govt. of India	Snakebite management as per Standard Treatment Guidelines (STG,2017) for snakebite management, Government of India
**ASVs utilization**	No uniform protocol followed for ASV administration	Medical Officers at PHC, RH and SDH administered recommended ASV dose as per STG
	ASV intra dermal skin test was reported as common clinical practice	ASV test dose practice is discontinued
**Management practices**	Medical Officers at PHCs did not manage snakebite envenomation cases due to fear of anaphylaxis reaction, lack of experience in snakebite management	Medical Officers are confident in management of snakebite envenomation at PHC level<br><br>Increase in ASV administration within golden hour period (< 1 hour of snakebite)<br><br>Reduction in referral cases to SDH, complicated cases were referred to SDH and higher centers

Capacity building of Public Health Department, Maharashtra State:

- The Commissioner, National Health Mission, Maharashtra State requested ICMR-NIRRH to provide technical support for capacity of building of Medical Officers in Maharashtra State. Accordingly, one-day training program on “Prevention, diagnosis and Management of snakebite envenomation and scorpion sting” was organized on 24th August 2018 at Pune, Maharashtra. A total of 59 Medical Officers working at PHCs, RHs and SDHs in Pune district were trained.
- Training modules for healthcare providers and IEC materials and copies of Standard Treatment Guidelines were provided to the Deputy Director Health Services, Pune for distribution in Pune District of Maharashtra State.

Research tools developed and validated for earlier study of MRHRU Dahanu will be used with appropriate modifications after National consultation meeting with snakebite experts and collaborating team.

Methodology:

Study Design: Implementation Research Project

Duration of Study: 24 months

- Preparatory phase (Revision and finalization of research tools, IEC, training manuals etc by Technical Advisory Group) : 6 months
- Total period which may be needed for collecting the data: 12 months
    - Prospective data collection from health facilities (June-September): 4 months
    - Data collection for impact evaluation (Objective 4) : 8 months
- Period that may be required for analyzing the data: 6 months

Study Sites:

Geographical region	State	Study site
West Zone	Maharashtra	Shahapur Block in Thane District<br>Aheri Block in Gadchiroli District
East Zone	Odisha	Khurda Block in Khurda District<br>Rayagada Block in Kasipur District

Sample Size: All ASHA/ANM/MPW, Medical Officers from the study area will be requested to participate in the study. A total of 24 Master Trainers (6 per study block) will be trained by National Experts Based on our earlier experience of snakebite study conducted in Dahanu block, each block will have approximately 40 Medical Officers; so for the proposed study total MOs undergoing training will be 160. Similarly, training will also be provided to outreach healthcare workers including ASHA, ANM, MPW. Approximately, 150 outreach healthcare workers per block will be trained so a total of 600 healthcare workers will be trained in 4 blocks. Actual numbers will vary and the same MOs or Health care workers may not be available throughout the study duration as there are frequent transfers of MOs and Healthcare staff due to various reasons.

Preparatory Phase (6 months)

Technical Advisory Committee (TAC) Members:

- Snakebite management experts (National and State)
- Program managers in MoHFW, Government of India and Public Health Department Maharashtra, and the Odisha States
- Herpetologists and Forest department representatives from the study area
- Researchers with experience in health system research, snakebite

- ICMR/DHR representatives

Activities and responsibilities of TAC:

- National consultation meetings for the development of IEC materials, training manuals, and research tools

Research Tools:

- Research tools will be revised based on the consultation meetings taking into consideration region-specific factors
- Culturally appropriate IEC materials will be developed in regional language

1. Retrospective data collection form
2. Focus Group Discussion (FGD) Guide
3. Interview questionnaire for ASHA/ANM/MPW to assess the knowledge gaps
4. Interview questionnaire for Medical Officers (PHC/CHC/DH)
5. Facility check survey forms (Infrastructure Assessment)

6. Training manuals for outreach health workers (ASHA, ANM, MPW) into regional languages
7. Training manuals for Master Trainers
8. Training manual for Medical Officers (Quick reference guide, Snakebite treatment flyer)
9. Retrospective data collection forms
10. Monitoring and evaluation checklist
11. Prospective snakebite management form

Objective I: To increase the awareness and empower the community on prevention, first aid, and early transport of snakebite patients to the nearest health facility for reducing the mortality and morbidity due to snakebite envenomation in selected districts of Maharashtra and Orissa

Baseline/retrospective data collection :
Two-year retrospective data will be collected from all the public health facilities in study areas. The data collection form will capture information on socio-demographic characteristics, the total number of snakebite cases (OPD/IPD), deaths due to snakebites, signs, and symptoms, first aid, and usage of ASV, treatment practices, etc wherever available from the hospital records. The data on snakebite deaths will also be collected from the municipal corporation records, gram panchayat, crematorium or any other institutions wherever available in the study areas to ensure that all deaths due to snakebite are captured in addition to data from hospital records.

Focus group discussions (FGDs): FGDs will be conducted in each study area. FGDs will be conducted separately for males and females. The selection of male and female participants for FGD will be as per the convenience of the participants.
Inclusion criteria:
1) Participants belonging to the community
2) Willing and able to actively participate
3) Above 18 years

Exclusion Criteria:
1) Participants not belonging to the community
2) Health workers directly and indirectly involved in snakebite management

In each block, we will conduct total 6 FGDs (3 males, 3 females). A total of 24 FGDs will be conducted but the number of FGDs will vary according to saturation of responses. Each FGD will be restricted to 8 to 16 participants.The selection of male and female participants for FGD will be as per the convenience of the participants. Study participants will be given a copy of participant information sheet and informed consent form shared and explained in vernacular language (Regional Language) to ensure voluntary participation. The research team will facilitate the discussion about perception, knowledge, awareness on snakes and snakebites, prevention methods, first aid practices, healthcare-seeking behavior of the community for snakebites. Audiotapes of the participant discussion will be recorded and notes will be taken. Themes and subthemes will be identified based on the transcripts and coding analysis will be carried out. Two or three trial FGDs will also be conducted to understand the community awareness before the study. This data will not be accounted for the analysis.

- Interview of outreach Health Care Providers Assessment (ASHA/ANM/MPW) to assess the knowledge gaps using a pre-tested and validated questionnaire.
- Interview of Medical Officers (PHC/CHC/DH) to assess the knowledge gaps using a pre-tested and validated questionnaire.
- Infrastructure Assessment (Facility check survey)

The developed questionnaire will be validated through, face validity, content, and construct validity. Any unclear questionnaire below 50% (percentage of correct responses overall) will be rewritten. The readability of the questionnaire will be assessed using the Simple Measure of Gobbledygook (SMOG) score.

Interventions:

IEC materials in local language for the community, snake rescuers (Sarpa Mitra/snake friends) school teachers, panchayat members, and traditional healers. The IEC materials will include the following information:

- Pictures of the venomous snakes commonly found in the study area
- Knowledge about venomous and non-venomous snakes
- Symptoms of snakebite envenomation in simple non-medical terms in regional language
- Prevention of snakebites and first aid treatment
- Nearest health facility for snakebite treatment (distance in kms, contact numbers of health facilities), ambulance services 102,108 information

The IEC materials will be displayed at Schools, Gram Panchayat in villages, Anganawadi, Tribal Residential Schools, Block panchayat Office, Sub Centers, PHCs, CHCs.

Community meetings and Public talks:

- ICMR snakebite project staff, and primary health care workers (ASHA, ANM and MPW) will be trained to educate community through community meetings for creating awareness on prevention, first aid and early transport of snakebite patients to nearest health facility

Retrospective data collection (2 years),
Focussed Group Discussions (FGDs) in community
IEC & Community meetings and public talks

⇩

Community Awareness, Education on risks, Prevention of Snakebite & Healthcare seeking behavior

⇩

Improved healthcare-seeking behavior, early referral for ASV administration

⇩

Reduction in snakebite mortality and morbidity

Objective II: To evaluate the component of training of the Medical Officers and other healthcare providers on management of snakebite and also understand the ASVs distribution and utilization (availability, factors restricting availability, quantity) in public health facilities in selected districts of Maharashtra and Orissa

Methods:

Interview of outreach Health Care Providers Assessment (ASHA/ANM/MPW) to assess the knowledge gaps using a pre-tested and validated questionnaire.

The questionnaire will broadly cover following points:

- Knowledge about venomous and non- venomous snakes in the study region
- Knowledge about symptoms of venomous poisonous bite and how to differentiate venomous snakebites from non-venomous snakebites
- first aid for snakebite and previous experience of giving first aid to snakebite patients
- Knowledge on ASV and emergency medical drugs for snakebite treatment

Interview of Medical Officers (PHC/CHC/DH) to assess the knowledge gaps using a pre-tested and validated questionnaire.

The questionnaire will broadly cover following points:

- Assessment of knowledge about venomous and non- venomous snakes in the study region
- Knowledge on about venomous snakebites and their clinical presentation, .and management
- Information on experience of snakebite management
- Information on training for snakebite management earlier
- Knowledge about referral criteria

- Knowledge and awareness on National snakebite management protocol (2009) and Standard Treatment Guidelines (STG,2017) or any other guidelines for snakebite management

Infrastructure Assessment (Facility check survey):

Assessment of Infrastructure (village/Sub center level):

- Availability of public transport/ambulance.
- Training of ASHA/ANM/MPW on identification of venomous snakebites
- Availability of first aid kits for snakebite patients
- Referral linkages to nearest health facility (PHC/CHC) for snakebite treatment
- Distance in Kms from Village/Sub centre to nearest health facility (PHC/CHC)

Assessment of Infrastructure at PHC/CHC/DH:

-Training of Medical Officers and other health care workers

-Availability, distributions and utility of Anti snake venoms (ASVs)

-Referral linkages to nearest higher health care facility for treating snakebite complications

-Distance in Kms from to nearest higher health care facility for management of snakebite

-complications

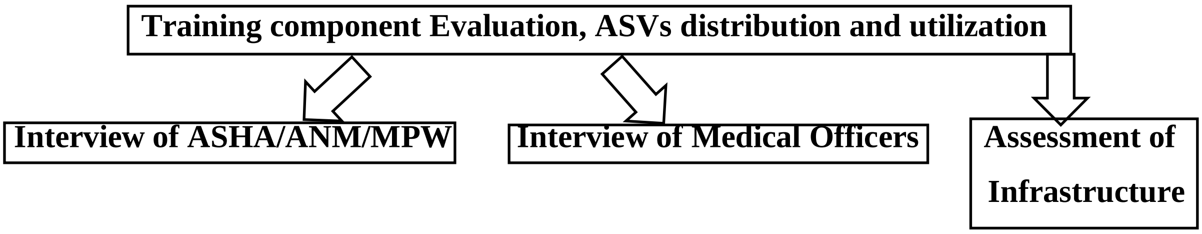

Objective III: To empower the health system for management of snakebite envenomation and optimal utilization of Anti snake venoms (ASVs) through implementation of Standard Treatment Guidelines (STG) of Government of India

Capacity building of Medical Officers (PHC, CHC, District Hospital):

-Team of National experts for snakebite management (Expert group STG, 2017) will provide training to master trainers and also provide technical support for regional training. [Dr Himmatrao Bawaskar and other experts who contributed for the development of STG, 2017 will provide training]

-Region wise snakebite experts will train the master trainers from each study site in Maharashtra and Odisha.

-Total 6 Master Trainers from each study site: 2 Master Trainers from State Public Health Department (Two Senior MOs from District Hospital and two MOs from CHC

, 2 Senior faculties Medicine Department of linked Medical College will be trained.

- Periodic training of all Medical Officers working in public health system in study area will be conducted every 6 months.
- All medical officers will be provided copies of Training modules prepared as per the Standardized Treatment Guidelines (STG, 2017)

  -*Quick reference guide*

  -*Snakebite treatment flyer*

Detailed Training will cover following aspects:

- When to suspect/recognize snakebite.
- Four presenting clinical syndromes of snakebites.
- First aid measures at health care facility.
- Signs and symptoms of snakebite envenomation.
- Patient assessment on arrival – critical, non-critical and critical after stabilization
- Examination of pregnant women.
- Laboratory investigations.
- Anti-snake venom therapy.
- Level specific management of snake bite and Referral criteria.
- Snake bite management at PHC, CHC, DH and higher facilities

Duration of Master Trainer training: 2 days

- Lecture and practical demonstration with exercises in small groups.

Snakebite training of MOs will be conducted every 6 months for 2 years

- Master Trainers will conduct the training of Medical Officers in their respective study area
- It will be mandatory for all Medical Officers working in study area
- Pre and post training evaluation of Medical Officers
- Assessment of Response to training

ASVs and essential medicines stock assessment every 3 months (PHC, CHC,) by the site investigators

Capacity building of outreach health staff (ASHA, ANM, MPW other healthcare providers**):**

- Periodic Training (every 6 months for 2 years) of health care providers will be conducted on snakebite prevention, diagnosis and management
- Training will also focus on first aid skills, immobilization techniques, cardiopulmonary resuscitation (CPR) etc.
- Medical Officers trained by Master trainers will conduct the training program for ASHA, ANM,

MPW and other healthcare providers at their respective health facilities.

- Training manuals for ASHA, ANM, MPW, and other healthcare providers developed by ICMR-NIRRH will be translated into regional languages
- Pre and post training evaluation to assess knowledge retention and assess impact of training

Quick reference charts as per STG (2017) on first aid and management of snakebite in English and regional languages will be displayed in OPD and indoor wards of PHC, CHC, in study area.

Monitoring and supervision of implementation of Standard Treatment Guidelines (STG, 2017) for snakebite Management.

- Periodic supervision at Sub centre, PHC, CHC to ensure snake management as per Standard Treatment Guidelines by a team of master trainers
- Notification of snakebite cases from villages, Sub centre, PHC to CHC Harbhanga Boudh District and CHC Jeypore Block of Koraput in Odisha.
- For Maharshtra state: Notification of snakebite cases to SDH Shahapur, Thane District and SDH Aheri in Gadchiroli
- Strict monitoring will be carried out to ensure that every snakebite patient is managed as per STG, 2017.
- The details of the snakebite treatment will be entered in a prospective data collection form signed by the head of the health facility. Project Staff will ensure the weekly collection of prospective data forms and data entry operator will enter the data regularly in a centralized database.
- Site Co- PI, Co-Is and Scientist C - Medical (project staff) will be responsible for monitoring the record of total number snakebite cases in each health facility.
- Details of Cases managed at PHC, CHC referred to higher centers (DH and/ Medical College) will be recorded
- Total number of deaths due to snakebite will be recorded before and after capacity building
- Treatment of severe snakebite envenomation and management of complications will be evaluated

Capacity Building of Medical Officers

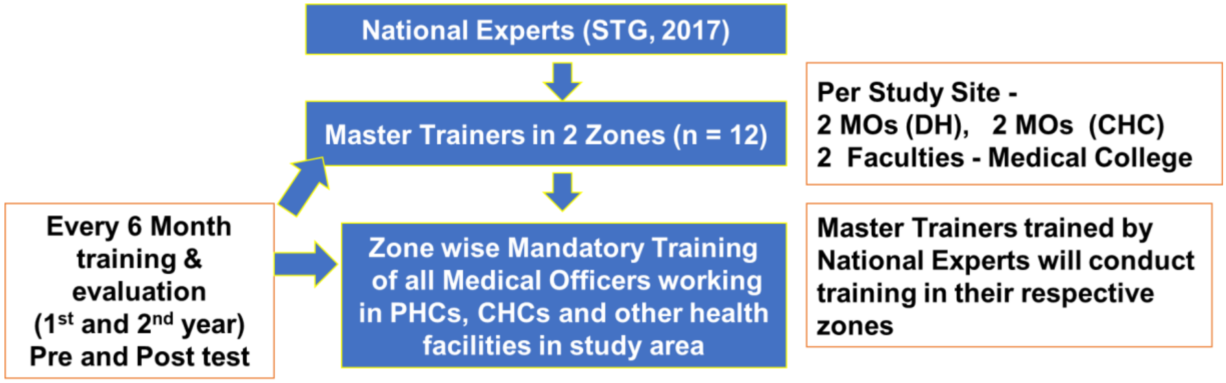

Capacity building of peripheral healthcare workers

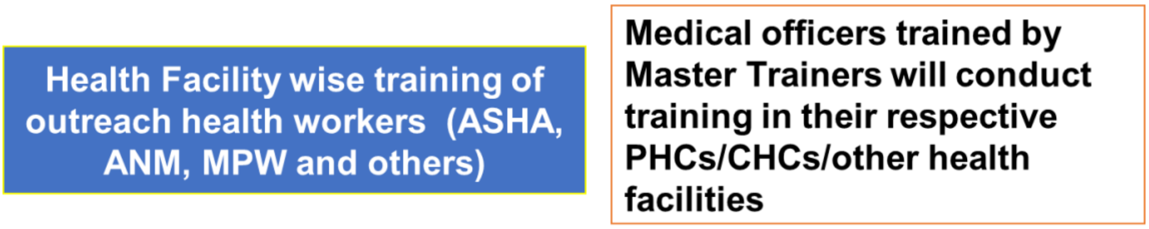

Objective IV: To study the impact of capacity building of health system on reducing the mortality and morbidity due to snakebite envenomation in selected districts of Maharashtra and Orissa

- The impact of capacity building will be measured by periodic data analysis
- FGDs will be conducted in the selected study sites to assess the impact of intervention
- Prospective data on snakebite management will be collected as per the format (Format already developed and implemented in earlier study)
- The information on immediate first aid and referral cases within golden hour (within 1 hour of snakebite)
- Total number of suspected snakebite cases where ASV was given within 1 hour of snake bite
- Reduction in time of snakebite and ASV administration will be analyzed
- Data on better management of snakebite cases by health care providers at different levels –

community/village level, sub centre-PHC-CHC

- Data on snake bite deaths and comparison with baseline data

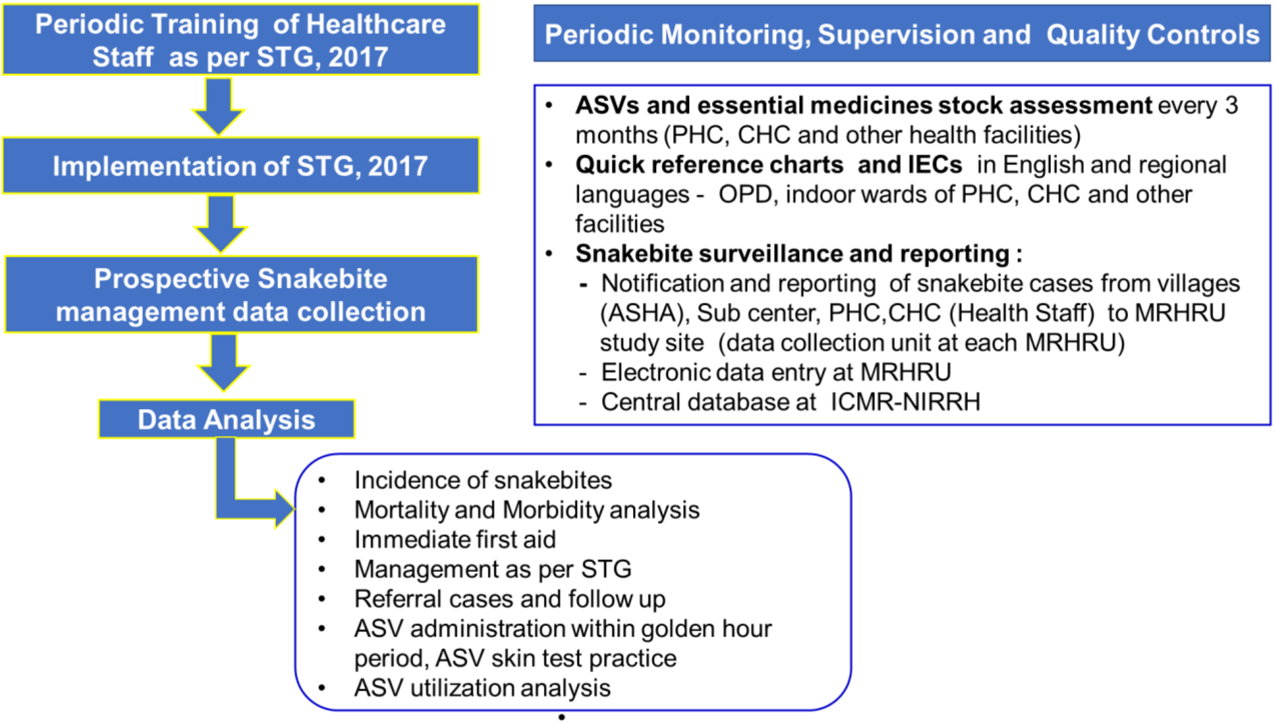

Relevance of research project to public health:

Expected outcome

a)Short term:

- The results of this study will be useful for finalization of National Snakebite Management Protocol relevant to public health system taking into consideration regional differences in India.

- The study will empower the community and health system for prevention and improved management of snakebites in selected regions in Maharashtra and Odisha.

- The results of the study will be crucial for establishing centers of excellence for snakebite management in Maharashtra and Odisha.

b) Long term:

- The results of the study will help in reducing the mortality and morbidity due to

snakebites in selected regions in India and also useful for phase II implementation in other states having high burden of snakebites.

- The evidence generated from the study may be useful to the Government of India for developing National Strategies to reduce burden of on snakebites.

- Model clinical snakebite management centers can be established all over country based on the outcome of the study

Bibliography

1 Gutiérrez JM, Calvete JJ, Habib AG, Harrison RA, Williams DJ, Warrell DA. Snakebite envenoming. *Nature Reviews Disease Primers* 2017; **3**: 17079.

2 Lancet T. Snakebite—emerging from the shadows of neglect. *The Lancet* 2019; **393**: 2175.

3 Mohapatra B, Warrell DA, Suraweera W, *et al.* Snakebite Mortality in India: A Nationally Representative Mortality Survey. *PLOS Neglected Tropical Diseases* 2011; **5**: e1018.

4 Majumder D, Sinha A, Bhattacharya SK, Ram R, Dasgupta U, Ram A. Epidemiological profile of snake bite in South 24 Parganas district of West Bengal with focus on underreporting of snake bite deaths. *Indian Journal of Public Health* 2014; **58**: 17.

5 Alirol E, Sharma SK, Bawaskar HS, Kuch U, Chappuis F. Snake Bite in South Asia: A Review. *PLOS Neglected Tropical Diseases* 2010; **4**: e603.

6 Bawaskar HS, Bawaskar PH, Bawaskar PH. Snake bite in India: a neglected disease of poverty. *The Lancet* 2017; **390**: 1947–8.

7 Chauhan V, Thakur S. The North–South divide in snake bite envenomation in India. *J Emerg Trauma Shock* 2016; **9**: 151–4.

8 Bawaskar HS, Bawaskar PH, Punde DP, Inamdar MK, Dongare RB, Bhoite RR. Profile of snakebite envenoming in rural Maharashtra, India. *J Assoc Physicians India* 2008; **56**: 88–95.

9 Bawaskar HS, Bawaskar PH. Call for global snake-bite control and procurement funding. *The Lancet* 2001; **357**: 1132–3.

10 Burki T. Resolution on snakebite envenoming adopted at the WHA. *The Lancet* 2018; **391**: 2311.

11 Minghui R, Malecela MN, Cooke E, Abela-Ridder B. WHO's Snakebite Envenoming Strategy for prevention and control. *The Lancet Global Health* 2019; **7**: e837–8.

12 Standard Treatment Guidelines - Governnment of India. http://www.nhm.gov.in/nrhm-instate/520-standard-treatment-guidelines.html (accessed June 4, 2018).

13 Gajbhiye R, Khan S, Kokate P, *et al.* Incidence & management practices of snakebite: A retrospective study at Sub-District Hospital, Dahanu, Maharashtra, India. *Indian J Med Res* 2019; **150**: 412–6.

14 Chaaithanya IK, Salvi N, Bhoye P, *et al.* Anti-snake Venom (ASV) Intradermal Skin Test, a Common Clinical Practice in the Primary Health Care Setting in Tribal Block of Dahanu, Maharashtra, India. *J Assoc Physicians India* 2020; **68**: 87–8.

15 Chaaithanya IK, Abnave D, Bawaskar H, *et al.* Perceptions, awareness on snakebite envenoming among the tribal community and health care providers of Dahanu block, Palghar District in Maharashtra, India. *PLOS ONE* 2021; **16**: e0255657.
