# Supplementary material for: National snakebite project on capacity building of health system on prevention and management of snakebite envenoming including its complications in selected districts of Maharashtra and Odisha in India: A study protocol
Source: PLoS One. 2023 Feb 17;18(2):e0281809. doi: 10.1371/journal.pone.0281809 (PMC9937454; doi:10.1371/journal.pone.0281809)
Supplement: Attachment — Submitted filename: Parliament Question Snakebite India.pdf [file pone.0281809.s006.pdf]

GOVERNMENT OF INDIA
MINISTRY OF HEALTH AND FAMILY WELFARE
DEPARTMENT OF HEALTH AND FAMILY WELFARE

LOK SABHA
UNSTARRED QUESTION NO. 1014
TO BE ANSWERED ON 7th FEBRUARY, 2020

SNAKE BITE DEATHS

1014. SHRI T.N. PRATHAPAN:

Will the Minister of **HEALTH AND FAMILY WELFARE** be pleased to state:

(a) whether as per media reports India is considered as the high burden country in terms of snake bite death/ injury/disability and the policies and actions are not adequate in this regard, if so, the details thereof along with recent snake bite and injury/disability reported, State/ UT-wise;

(b) the steps taken/proposed to address the issues related to reporting/non-reporting of the snake bite death/ injuries/disability and to implement the recent WHO's global strategy for prevention and control of snake bite envenoming in India;

(c) whether the Government is considering a national strategy of snake bite envenoming to control, prevent and address the India specific situation and if so, the details thereof;

(d) whether the Government is aware of some studies on quality of the Anti-Snake Venom (ASV) and its effectiveness in mitigating snake bite and the WHO standards not being followed in ASV currently manufactured in India in terms of collection, storage, transport and purification and if so, the details thereof;

(e) whether low price fixed for Indian ASV by DPCO is hampering its quality and putting lives in danger, if so, the details thereof; and

(f) the measures taken/proposed to improve the manufacturing standard and quality of ASV that is representative of snake species all over the country?

ANSWER
THE MINISTER OF STATE IN THE MINISTRY OF HEALTH AND FAMILY WELFARE
(SHRI ASHWINI KUMAR CHOUBEY)

(a): As per data available with Central Bureau of Health Intelligence (CBHI), under this Ministry the State/UT-wise details of cases and deaths reported due to snake bites, during the last three years in the country are given in annexure.

(b) & (c): Indian Council of Medical Research (ICMR) has constituted a "National Task Force for Research on Snake Bite in India" with the following national level multi-centric projects:

I. Nationwide Study to estimate incidence, mortality, morbidity and economic burden due to snakebite in India

II. ICMR National Snakebite Project (INSP) on capacity building of health system on prevention and management of snakebite envenomation including its complications.

Ministry of Health and Family Welfare has also issued directions to all the States/UTs to includes Anti Snake Venom Serum (ASVS) in the list of essential drugs of the State, procure these drugs locally for supply to Health Facilities for use to the needy persons and support the same under National Health Mission.

(d): As per information available in public domain, one study has indicated that currently marketed anti-venoms were found to exhibit poor dose efficacy and venom recognition potential against the venom of neglected snake species while the marketed anti-venom formulations are meant for the commonly found four snake species. The snake venom antiserum is required to be manufactured in India as per the provision of the Drugs and Cosmetic Act, 1940 and rules framed thereunder.

(e) & (f): The drug manufacturers in the Country are required to comply with the standards laid down in the Indian Pharmacopoeia and the provisions of the Drugs & Cosmetics Act, 1940. As per the rules framed under the Drugs & Cosmetics Act, 1940, the applicants of drug manufacturing units have to mandatorily submit evidence of stability, safety of excipients, etc. to the State Licencing Authority.

Government have taken measures to ensure quality of the Drug, such as, amendment in the Drugs and Cosmetics Act, 1940 to provide stringent penalties for manufacture of spurious and adulterated drugs including making some offences cognizable and non-bailable, the testing capacities of Central Drugs Testing Laboratories under Central Drugs Standard Control Organization (CDSCO) are strengthened to expedite testing of drug samples in the country, joint pre-inspection of manufacturing units before grant of licence, joint inspection of manufacturing units to ensure compliance with the conditions of license and the provisions of the Drugs & Cosmetics Act and rules as per risk based approach, etc.

Annexure

State/UT-wise Cases and Deaths due to Snake Bite during the years 2016 – 2018

S. No.	State /U.T	2016		2017		2018 (Provisional)	
		Cases	Deaths	Cases	Deaths	Cases	Deaths
1	Andhra Pradesh	20,809	28	20,953	85	25,964	117
2	Arunachal Pradesh	43	0	37	0	13	1
3	Assam	265	0	810	0	2,318	0
4	Bihar	3,334	11	2,848	14	2,924	31
5	Chhattisgarh	3,533	60	4,109	52	4,148	33
6	Goa	620	1	1,189	8	1,025	1
7	Gujarat	5,872	72	6,209	49	7,064	50
8	Haryana	814	4	944	6	977	7
9	Himachal Pradesh	1,587	23	1,465	9	2,105	30
10	Jammu & Kashmir	1,357	0	1,422	1	1,391	1
11	Jharkhand	749	15	788	2	481	2
12	Karnataka	9,730	46	8,084	28	7,507	30
13	Kerala	3,019	12	3,608	13	4,189	11
14	Madhya Pradesh	5,010	113	5,346	96	2,646	39
15	Maharashtra	29,629	67	16,708	35	18,707	32
16	Manipur	118	0	150	0	50	1
17	Meghalaya	405	0	282	2	327	0
18	Mizoram	205	0	278	0	368	0
19	Nagaland	106	0	136	0	245	0
20	Odisha	15,405	120	15,274	147	8,003	98
21	Punjab	1,511	8	918	3	1,421	4
22	Rajasthan	4,929	32	6,720	45	5,810	30
23	Sikkim	242	2	130	0	117	1
24	Tamil Nadu	16,277	44	13,668	38	14,027	50
25	Telangana	13,729	96	2,975	2	3,306	9
26	Tripura	430	1	779	1	813	2
27	Uttarakhand	194	3	128	0	208	2
28	Uttar Pradesh	6,640	118	7,448	128	7,623	73
29	West Bengal	25,481	138	31,072	268	36,229	203
30	A & N Islands	294	0	195	1	208	5
31	Chandigarh	3,089	36	1,294	9	829	14
32	D & N Haveli	609	9	626	7	733	4
33	Daman & Diu	51	0	99	0	130	0
34	Delhi	200	4	263	2	168	1
35	Lakshadweep	0	0	0	0	0	0
36	Pondicherry	2,147	5	1,695	9	1,957	3
**Total**		**1,78,433**	**1068**	**1,58,650**	**1060**	**1,64,031**	**885**
