# Supplementary material for: National snakebite project on capacity building of health system on prevention and management of snakebite envenoming including its complications in selected districts of Maharashtra and Odisha in India: A study protocol
Source: PLoS One. 2023 Feb 17;18(2):e0281809. doi: 10.1371/journal.pone.0281809 (PMC9937454; doi:10.1371/journal.pone.0281809)
Supplement: Attachment — Submitted filename: Response to Reviewers.docx [file pone.0281809.s007.docx]

**RESPONSE TO REVIEWER’S COMMENTS**

**PONE-D-22-12125R1**

**Title:** National snakebite project on capacity building of health system on prevention and management of snakebite envenoming including its complications in selected districts of Maharashtra and Odisha in India: a study protocol

**Journal requirement**

1. Please ensure that your manuscript meets PLOS ONE's style requirements, including those for file naming. The PLOS ONE style templates can be found at
   <https://journals.plos.org/plosone/s/file?id=wjVg/PLOSOne_formatting_sample_main_body.pdf> and
   <https://journals.plos.org/plosone/s/file?id=ba62/PLOSOne_formatting_sample_title_authors_affiliations.pdf>

**Response:** The manuscript has been revised to meet PLOS ONE’s style requirements as per the templates provided in the comment.

Paragraph formatting and font size change are done throughout the manuscript.

Title and author page revisions on page no. 1-2

2. Thank you for stating the following in the Acknowledgments Section of your manuscript:
   “The authors also thank the Public Health Departments in Maharashtra and Odisha states for providing administrative permissions and support for the study. Dr. Rahul K Gajbhiye is an awardee of the DBT-Wellcome India alliance clinical and public health intermediate fellowship (Grant no. IA/CPHI/18/1/503933).”

   We note that you have provided additional information within the Acknowledgements Section that is not currently declared in your Funding Statement. Please note that funding information should not appear in the Acknowledgments section or other areas of your manuscript. We will only publish funding information present in the Funding Statement section of the online submission form.

   **Please remove any funding-related text from the manuscript** and let us know how you would like to update your Funding Statement. Currently, your Funding Statement reads as follows:

   “This project is funded by the Indian Council of Medical Research (ICMR), (no: 58/6/NTF-Snakebite/2019-NCD-II). The funding agency has no role in study design, collection, management, analysis and interpretation of data; writing of report; and the decision to submit the report for publication. The funding agency will have no authority over any of these activities.”

   **Please include your amended statements within your cover letter**; we will change the online submission form on your behalf.

**Response:** Funding-related text has been removed from the manuscript (Page no. 20-21, Acknowledgement section).

The amended funding statement has been included in the cover letter.

3. Thank you for stating the following financial disclosure:

   “This project is funded by the Indian Council of Medical Research (ICMR), (no: 58/6/NTF-Snakebite/2019-NCD-II). The funding agency has no role in study design, collection, management, analysis and interpretation of data; writing of report; and the decision to submit the report for publication. The funding agency will have no authority over any of these activities”

   Please state what role the funders took in the study. If the funders had no role, please state: "The funders had no role in study design, data collection and analysis, decision to publish, or preparation of the manuscript."

   If this statement is not correct you must amend it as needed.

   **Please include this amended Role of Funder statement in your cover letter**; we will change the online submission form on your behalf.

**Response:** The amended funding statement has been included in the cover letter. Manuscript Page no. 21, Line no. 454-457

4. We note that you have stated that you will provide repository information for your data at acceptance. Should your manuscript be accepted for publication, we will hold it until you provide the relevant accession numbers or DOIs necessary to access your data. **If you wish to make changes to your Data Availability statement, please describe these changes in your cover letter** and we will update your Data Availability statement to reflect the information you provide.

**Response:** The amended data availability statement has been included in the cover letter. Manuscript Page no. 23, Line no. 501-504

5. Your ethics statement should only appear in the Methods section of your manuscript. If your ethics statement is written in any section besides the Methods, please move it to the Methods section and delete it from any other section. Please ensure that your ethics statement is included in your manuscript, as the ethics statement entered into the online submission form will not be published alongside your manuscript.

**Response:** The ethics statement has been moved to the Methods section of the manuscript as per the comment.

Page No. 15-16, Line No. 329-341

**Reviewer #1:**

6. I thank the authors for carefully considering my comments on the prior draft. **The expanded statistical methods section is noted and I believe is sufficient for a study protocol.** Statistical methods sections are hard to write in protocols because (1) it's hard to predict what will be needed for analyses and (2) detail is needed to understand the proposal but too much detail may result in protocol revisions.

**Response:** We thank the reviewer for critically reviewing the statistical analysis section and providing constrictive feedback to improve it.

7. Regarding criterion 5, I see the changes to the study setting paragraph, but based on the tracked changes version of the manuscript I'm not convinced that the authors reviewed the whole manuscript in detail. I'd expect to see small changes throughout the manuscript. That said, maybe these were not recorded and, as I said prior, the article is intelligible, which is most important.

**Response:** The manuscript was thoroughly revised to make it intelligible and small typos and writing errors were corrected after reviewer’s feedback.

**Reviewer #2:**

8. The authors listed underestimated reports and deaths in consequence of snakebite envenoming, especially in Maharashtra state, India. However, to build a national snakebite project for strengthening health system on prevention in management, one important component is a reliable epidemiological surveillance system. **In this matter, no mention was given to snakebite envenoming compulsory notification and strategies to collect robust surveillance data**. Considering, as authors referred, that only 10% coverage of the actual snakebite burden have being captured by the official data (ref.10], it would be a crucial element of the project to increase the capillarity of the system to collect snakebite envenoming cases. The morbimortality data, as well as risk factors analysis of deaths and disabilities should be the basis to guide program interventions and sharpen public communications. Thus, **which strategies would be designed to generate sufficient quality data that matches the objectives of the protocol?**

**Response:** We thank the reviewer for raising the concern. The proposed study focuses on capacity building of health systems on prevention and management of snakebite envenomation and would be useful for updating the national protocol for snakebite treatment in India and providing regional inputs for the same.

Regarding the objectives of the present study, strategies to generate quality data, including validation of research tools, review and approval from Technical Advisory Committee, training of project staff before study implementation, IEC material dissemination, focus group discussions, health facility assessments are provided in sufficient detail in the protocol.

We agree with the reviewer about the pressing need to have a robust surveillance system to capture maximum cases and deaths due to snakebite. However, that remains beyond the scope of the present study. Community empowerment using culturally appropriate Information, Education and Communication (IEC) material is an integral component of the present study. Improving knowledge of the communities regarding the prevention and first aid of snakebite along with advocating healthcare seeking from trained medical practitioners is indirectly expected to improve their healthcare seeking behavior and contribute to improving the number of cases and deaths being reported at the health facilities in the study sites. Through training programs, the study also aims to empower the medical doctors and healthcare workers for the better prevention, diagnosis and management of snakebite envenomation. Availability of trained staff is also expected to improve the confidence that people have in the health systems and motivate them to seek appropriate care.

The ICMR task force project by Menon et al (doi.org/10.1371/journal.pone.0270735), have proposed a community-level surveillance for snakebites covering 31 districts in 13 states of India including Maharashtra to obtain the annual incidence of snakebites from the community. This study would help in building up a strong surveillance system in India and provide morbimortality data of snakebite envenomation. A significant difference, if any, in the findings of the Menon et al., study and the health system data can further pave way for recommending compulsory notification of snakebite envenomation in India, as suggested by the reviewer.

9. Phase I was described as been composed of retrospective data collection from public health facilities, and focal groups discussions in the community. **It is not clear the selection criteria for the health facilities. Would be at random? How representativeness will be guaranteed to assure that different categories of health facilities would be represented.**
   **Still in Phase I, how focal groups will be selected in the community to participate in the study? Community leaders? Family members of a snakebite patient? Neighbors? Extended family?**

**Response:** In the proposed study, we are going to include all the public health facilities in the study areas where snakebite management is expected to be provided. This includes all the Primary Health Centres, Rural Hospitals / Community Health Centres, Sub District Hospitals, District Hospitals. Medical Colleges where critical cases are referred from the study sites will also be included to ensure quality retrospective data collection. This has been included in the revised manuscript (Page no. 12-13, Line no. 250-255).

Additional data on snakebite deaths will also be collected from local authorities, panchayat office, and registrar of births and deaths so that the maximum number of deaths are captured. This section is already present in the manuscript (Page no. 13, Line no. 255-256).

Regarding selection of focal groups in the community, the selection process has been revised in the manuscript (Page no. 13, Line no. 264-269)

10. Pre and post-training evaluation will be done to assess knowledge but it is not clear how long before and after the distribution of educational materials distributed in key places. Another question is how participants will be selected; **will it be at random?**
    **Methodology of the intervention does not supply sufficient information to understand how deep will the authors explore this issue.**
    **Validation of the educational tools seems not to be included.**

**Response:** The study proposes to do a post-intervention evaluation after two months (Page 15, Line no. 320-322). The methodology including the selection of participants for the post-intervention evaluation will remain the same as that of pre-intervention assessment. The educational intervention would be implemented in the study sites by trained and experienced research staff. All the interventional material will be reviewed and endorsed by the technical advisory committee of the study. This has been added in the revised manuscript (Page no. 14, Line no. 280-282).

Mock or trial intervention procedures will be implemented to know the community's understanding capacity accordingly further intervention procedures will be carried out. This has been added in the revised manuscript (Page no. 13-14, Line no. 278-280).

Education tools will be validated with the help of the Technical advisory committee and local herpetologists and subject experts. This has been added in the revised manuscript (Page no. 14, Line no. 280-282).

We thank the editoral board, editor and reviewes for giving an opportunity to revise the manuscript.
